# Informed Consent in Clinical Training: Perspectives from Medical Students and Faculty in Portugal

**DOI:** 10.3390/healthcare12181818

**Published:** 2024-09-11

**Authors:** Carolina Frade Moreira, Cristina Costa-Santos, Bárbara Frade Moreira, Rui Nunes, Ivone Duarte

**Affiliations:** 1 Faculty of Medicine, University of Porto, 4200-319 Porto, Portugal; carolinafmoreira@outlook.pt; 2 Department of Community Medicine, Information and Health Decision Sciences (MEDCIDS)–Technology and Services Research–CINTESIS @ RISE, Faculty of Medicine, University of Porto, 4200-319 Porto, Portugal; csantos.cristina@gmail.com; 3 Department of General and Family Medicine, Family Health Unit Caminhos do Cértoma, Coimbra Local Health Unit, 3050-428 Pampilhosa, Portugal; barbarafmoreira@outlook.com; 4 Center of Bioethics, Faculty of Medicine, University of Porto, 4200-319 Porto, Portugal; ruinunes@med.up.pt

**Keywords:** informed consent, patients’ consent, clinical training, medical students, medical teachers, medical education, medical ethics

## Abstract

The student–patient relationship represents the cornerstone of medical education, shaping future doctors’ knowledge, skills and attitudes. While most patients allow student involvement in their care, some may express discomfort. Thus, obtaining explicit consent is essential to respect patients’ right of autonomy. This study mainly aims to assess the practical application of informed consent by medical students and teachers regarding students’ presence and participation in patients’ care. An observational cross-sectional study was performed, and an online questionnaire was given to students and teachers at a single medical school, via institutional email. The study included 289 participants, namely 232 students and 57 teachers. While 81% of teachers reported always asking the patient’s consent for students’ presence, only 28% of students claimed this to be the case. Despite challenges like overcrowding and limited time, involving students in healthcare benefits both students and patients. Moreover, medical ethics education is crucial to foster compassionate care and promote ethical reasoning. The disparities found between teachers’ practices and students’ perspectives highlight the need to intervene and provide them with an adequate education on ethical values in clinical practice. Strategic interventions at institutional levels are required for a simultaneous high quality of patient care and clinical training.

## 1. Introduction

Direct interaction with patients constitutes a key feature of medical curricula, leading to the development of knowledge, clinical reasoning, communication skills, and professional attitudes among medical students [1,2,3]. As a matter of fact, student–patient interactions at the bedside, guided by physicians during teaching sessions, continue to serve as the cornerstone for the comprehensive training of future doctors and the practice of medicine [2].

Throughout much of the history of Western medicine, paternalism prevailed, leaving patients with minimal control over their own medical treatment. However, in recent years, there has been a significant shift towards prioritizing patient autonomy [4], as paternalism and the passive patient gradually disappear [5]. Simultaneously, over the past decades, medical ethics has gained a growing emphasis within medical education institutions and among ethics educators, making it an essential component of medical education [6,7].

Patients are entitled to receive information regarding the nature of their current condition, the objectives of treatment, and the specific risks and benefits associated with alternative treatment options [8]. The process of informed consent takes place when communication between a patient and physician leads to the patient’s authorization or agreement to undergo a particular medical intervention [9]. For this reason, obtaining consent holds significant importance, serving as a fundamental component in showing respect for patients and their autonomy, promoting rapport, cultivating trust and nurturing a constructive doctor–patient relationship [10].

In the realm of healthcare, informed consent has evolved into an act of both legal and ethical-deontological significance [11]. From an ethical perspective, getting informed consent is good medical practice and the training of students is not an exception. Undeniably, a lack of valid informed consent represents a breach of proper medical practice, potentially leading to disciplinary, civil or criminal repercussions for the physician responsible [12].

It is important to mention that medical students, like clinical teachers, can be liable for damages caused to the patient, including those arising from infringement of the right to self-determination. Indeed, from a legal perspective, a failure to provide prior information to the patient and secure valid informed consent can be viewed as a factor contributing to liability for healthcare professionals [11].

The fundamental norm in the Portuguese legal system concerning the duty to inform is found in Article 157 of the Penal Code, which provides that: “Consent is only effective when the patient has been properly informed about the diagnosis, nature, scope, extent, and possible consequences of the intervention or treatment…” [13]. If there is a violation of this obligation, consent is ineffective, and thus the entire medical intervention is considered unlawful. If it cannot be proven that the duty to inform was fulfilled (except in special circumstances such as in emergencies), the responsibility for the medical intervention, as well as its failures, uncontrollable side effects, and other damages resulting from the intervention, fall upon the doctor [14].

Equally important is the patient’s right to dissent. According to the Oviedo Convention, any intervention can only be carried out after obtaining free and informed consent, and patients can, at any time, freely withdraw their consent [15]. This way, patients can change their decision and their consent can be revoked without being subject to any formality [16].

In terms of medical education, explicit consent for student participation in healthcare is crucial as it ensures that patients are aware of who will be involved in their care and the motives behind it [10]. Informed consent in medical education implies explicitly informing the patient about the participation of medical students in treatment and care, ensuring the patient comprehends the level of knowledge, skill and experience possessed by these students, and obtaining the patient’s voluntary agreement to their involvement in treatment and care [17].

When visiting a teaching hospital in Portugal, patients are generally aware of the possibility of being asked whether they consent to medical students participating in their healthcare. However, there is no Portuguese legislation that obliges the patient, even if in a university hospital, to be observed by a medical student. According to Portuguese law, every medical intervention should only proceed after obtaining consent, with established codes of conduct and ethical guidelines specifically designed for medical students [12].

According to the literature regarding medical students, especially during the clinical years, they experience a decline in their capacity to identify ethical dilemmas and to address them with empathy and compassionate reasoning [6,18]. Moreover, as they progress through their clinical training years, their sense of responsibility to inform patients about their student status tends to diminish [19]. Actually, the possibility of a patient’s refusal and rejection may exacerbate students’ lack of confidence, which could be a reason why students still hesitate to fully disclose their levels of experience [20]. Additionally, interactions between patients and medical students frequently take place in bustling settings, where clinical staff are under pressure, patient turnover is rapid and opportunities to request consent are limited [21].

For these reasons, it becomes vital to incorporate and integrate behaviors and attitudes essential for ethical healthcare practice, from their initial practical experiences in classes and throughout students’ educational journey [22]. In fact, the primary goal of medical ethics education is to cultivate dedicated physicians who are adequately prepared to recognize and analyze ethical dilemmas encountered in clinical settings. Ethics education should empower students to identify ethical challenges, approach them with rationality, and respond responsibly [23].

On the other hand, medical educators must consistently acknowledge the importance of instilling in young physicians the principles of ethical medical practice. The literature emphasizes the critical role of leading by example, advocating for medical students to cultivate empathy through the guidance of trustworthy role models rather than mere lectures. With positive reinforcement from such mentors, the front line of ethics education for medical students stands a greater chance of success [18]. Thus, both students and their clinical teachers are required to obtain meaningful informed consent from patients or their legal representatives, so they can be involved and participate in clinical teaching [5].

What remains vital is safeguarding the involvement of learners on the care team, cultivating future generations of physicians, and upholding transparency regarding their role in discussions with patients in order to preserve patient autonomy [24]. Therefore, there is a delicate and complex balance between safeguarding patients, minimizing distress for learners and guaranteeing comprehensive education [25].

Therefore, this study aims to describe the conduct of medical students and their respective teachers relating to the practical application of informed consent, and their perspectives on students’ presence and participation in patients’ care. As a secondary aim, this study intends to compare the students’ experience by attending year of medical school. In addition, this article can alert to the possible need for a future intervention in reshaping and reinforcing these values within medical education, aiming to simultaneously improve the quality of both patient care and medical training standards.

## 2. Materials and Methods

This study was conducted between March and June of 2019, at the Faculty of Medicine of the University of Porto (FMUP), Portugal.

An observational cross-sectional study was performed using an online questionnaire developed for medical school teachers with a tutoring role in FMUP and medical students attending the fourth, fifth and sixth years of medical school, also in FMUP, and distributed via institutional email, insisting upon using the same method in case of no response and with a convenience sample size. Involvement in this activity was entirely voluntary and consent was tacit by answering and sending the completed questionnaire.

The questionnaire given to teachers consisted of two sections: the first one collected sociodemographic data (gender, age, academic qualifications, specialty, years in practice and years of tutoring), while the second section contained questions pertinent to the scope of the research project. Similarly, the students’ survey comprised two parts: the initial part gathered sociodemographic information (gender, age and attending year of medical school), and the following part addressed inquiries relevant to the research project’s objectives. Each questionnaire lasted approximately 5 min.

The questionnaire was not formally validated. However, it was designed based on a literature review and expert input to ensure it accurately captures the dimensions of interest.

This study was approved by the Ethics Committee of the University Hospital Centre of São João and Faculty of Medicine of the University of Porto, on 28 December 2018 (ref. 392/18).

Data were analyzed using Statistical Package for Social Sciences (SPSS), and results were described with absolute and relative frequencies. Comparison by student’s academic year was carried out using chi-square or Fisher’s exact tests as appropriate, and a significance level of 5% was considered statistically significant.

## 3. Results

The sample consisted of 289 participants that were interviewed, namely 232 students and 57 teachers.

Of the 232 students who responded to the questionnaire, 157 (68%) were female and 75 (32%) were male. The median age of the students was 23 years (minimum of 20 and maximum of 39 years); 92 students (40%) were in the fourth year of medical school, 65 (28%) in the fifth year, and 75 (32%) in the sixth year.

Concerning the 57 tutors who responded, 21 (37%) were female and 36 (63%) were male, with a mean age of 53 years (standard deviation of 10 years). Of the 57 tutors who responded, 45 (79%) held a PhD, 4 (7%) a master’s degree, and 8 (14%) a bachelor’s degree. The vast majority (88%) of tutors had been practicing their profession for 10 years or more. 54% of them have been serving as tutors for more than 15 years, 26% between 11 to 15 years, 11% between 5 to 10 years, and 9% for less than 5 years.

Among the 232 students interviewed, 86% always presented themselves as a student to patients, and 53% mentioned the year of the course they were in, when presenting themselves. The majority of the students (69%) considered the consultation dynamics, under current conditions, to be less adequate or even inadequate for the acquisition of competences. The main limitations to the consultation dynamics presented by the students were: many consultations, limited time and physical space, excess of students, lack of privacy, little time for discussion with clinicians and lack of possibility of interaction with the patient. In addition, 16% of students reported not knowing the National Code of Ethics for Medical Students.

Of the 72% of students who said their teachers did not always request consent from the patient for the students to be present during the consultation, some cited the following circumstances in which this occurs: forgetfulness; only if it is necessary to gather the patient’s medical history; if there is a physical examination that does not require the patient to remove clothing; consultations that are not considered “sensitive”; incapacitated patients who are unable to give consent; patients who do not question the presence of the students and therefore are assumed not to mind; patients who were previously in the presence of students; the belief that patient participation is implied, given it is a teaching hospital; when there are many patients/lack of time; when students are already in the room; when it is the last consultation.

When asked about how many students, on average, usually attend the consultation, the students’ response was a median of 4 (minimum 1 and maximum 7), with 23% of them considering this number to be very uncomfortable for the patient (Table 1).

Concerning the teachers, 81% always asked the patient for consent to be in the presence of students, with 49% who did so when the students were already present. In addition, 71% of them considered the consultation dynamics, under current conditions, to be less adequate or even inadequate for the acquisition of competences. The main limitations to the dynamics of consultation presented by the teachers were: excess of patients, lack of time, reduced physical space, high number of students and damage to the doctor–patient therapeutic relationship. Additionally, more than half of teachers (53%) reported not knowing the National Code of Ethics for Medical Students.

Furthermore, there was a significant difference between students’ answers regarding whether consent is asked before they enter the room or in their presence, depending on the academic year (*p* = 0.026): final year students were those who reported the highest percentage of teachers requesting patients’ consent in their presence (88%) (Table 2). Moreover, there are also significant differences in whether students discuss with their teachers using medical jargon in the presence of patients (*p* < 0.001), as sixth-year students reported a lower frequency of discussing patients’ clinical status with teachers in their presence (19% versus 34% and 48% for sixth year, fifth year and fourth year, respectively) (Table 2).

## 4. Discussion

As medical students progress from beginners to junior doctors, engaging with patients becomes an increasingly vital aspect of their learning journey. However, prior to participating in any aspect of a patient’s care, they must obtain the patient’s consent [21]. “Consent” denotes a voluntary agreement and is necessary for procedures such as history taking and physical examination, and it can be implied or expressed either orally or in writing [20].

The right to informed consent is an axiological and normative postulate recognized by many legal systems and, undoubtedly, enshrined in Portuguese law. In 1981, the Declaration of Lisbon on the Rights of the Patient by the World Medical Association proclaims: “The patient has the right to accept or refuse treatment after receiving adequate information” [14].

The dual nature of the concept of informed consent has become an undeniable reality that requires our attention. While, from a clinical standpoint, it signifies a moment of dialogue and mutual understanding between the physician and the patient, forming the cornerstone of a conscientious care relationship, it is also crucial to recognize that the doctrine of informed consent solidified within the legal domain [11].

That being said, students and teachers should not obtain informed consent simply because it is required by law, but because they believe it is the ethically and morally correct attitude. Rather than viewing informed consent as just another legal or ethical obligation, students and teachers should seize the opportunity to respect the patient’s rights while also providing them significant benefits and contributing to the development of the students’ professional attitudes [17].

Studies show that, in reality, patients are rarely informed of the role of students in their medical care [19] and, although only a small percentage of medical students introduced themselves as “doctor” all of them had encountered situations where other healthcare team members referred to them as “doctor” [26]. Still, contrary to the literature, this study found that the consistent practice of the majority of students included disclosing their student status to patients. Despite this, the study also revealed a disparity concerning the level of detail provided by students during introductions, with just over half mentioning their current academic year.

However, the importance of disclosing the learner’s role lies in the fact that non-medical individuals may not always distinguish between a medical student and a board-certified specialist [20,24]. In fact, students who are proactive in introducing themselves and clarifying their status are more inclined to obtain specific consent, regardless of the invasiveness of the procedure [26].

On the other hand, the fact that, in academic hospitals, students may provide care to the patients can be seen as an opportunity to improve patient–student communication and uphold patient autonomy, by ensuring transparency regarding the roles of individuals within the care team [24]. Therefore, it is important that both teachers and students always be mindful of the considerations related to informed consent and preserving the dignity of the patient [27], as incoming patients need to be well-informed about the nature of the teaching hospital upon admission [20]. By honoring patients’ autonomy and demonstrating responsibility towards their rights, patients are more likely to participate in the teaching process involving medical students. This, in turn, ensures that patients receive a standard level of care provided by competent staff [1].

In this study, a significant portion of students reported inadequate consultation dynamics for acquiring skills, mentioning motives such as an excess of students and little time for discussion with clinicians, as well as a lack of patient interaction opportunities. Therefore, these factors can represent great obstacles in students’ clinical training setting. One possible approach to solve such obstacles is to reduce the student/patient ratio during interactions, enhancing both patient comfort and students’ learning experience [3]. It could be beneficial for students to engage with patients both before and after consultations. Beforehand, ensuring patients grasp the educational nature of their involvement is crucial, while afterward, discussing whether educational objectives were met is valuable. This approach not only enhances medical students’ education by fostering an understanding of patients’ perspectives and beliefs but also initiates their learning about collaborating with patients, which is vital for their future practice [5].

Despite that, the involvement of medical students in procedures at academic medical centers is essential for medical education [24]. Physical examination, for instance, is a fundamental aspect of delivering high-quality clinical care, and it is imperative that future generations of physicians have the opportunity to refine their knowledge and skills in this area [25]. Due to the limited experience of students [26], all procedures and interventions in which students participate are conducted under the supervision of an attending physician [24]. Undoubtedly, students stand to gain valuable experience by practicing skills on patients, and, reciprocally, patients are also believed to benefit from the focused attention provided. The involvement of medical students offers patients an additional opportunity to discuss concerns and receive information, since students often have significantly more time available to spend with patients when obtaining a medical history [26].

As medical students proceed through training, professional and moral developments remain vital aspects of being a physician [28]. Nevertheless, according to the literature, engagement in clinical rotations diminishes the perceived importance of obtaining informed consent in the eyes of the students [29]. By contrast, this study revealed that concerning students’ presentation and disclosure of their current academic year to patients, this practice was quite consistent throughout the fourth, fifth and sixth years.

In recent decades, various transformations in the healthcare system have fostered an environment where students often feel compelled to set aside their inclination to advocate for patients, primarily to conform to the hierarchy within the medical team and achieve favorable grades [18]. However, this study showed different findings, as final year students reported a lower frequency of technical discussions, that is, using medical jargon, with their teachers in the patient’s presence. In the authors’ opinion, this could be due to the fact that more mature students realize that discussions with technical terms may not be understood by patients, making them feel excluded from the dialogue.

However, this study also showed that over a quarter of students were unaware of the National Code of Ethics for Medical Students, indicating potential and important gaps between ethical understanding and practice. Given these data, it is crucial to increasingly acknowledge the harmful impacts of the hidden curriculum, while recognizing the imperative to foster the development of ethical values among medical students [30].

Teaching ethics represents a method for cultivating virtuous physicians, as it equips them with the necessary skills to analyze and resolve ethical dilemmas. Medical ethics education ought to be seamlessly integrated across the entirety of medical school curricula, and perceived as an ongoing process [7]. The unique contribution of an ethics curriculum is to instill a component of moral growth, specifically the ethical reasoning essential for professional development [28].

However, the curricula of medical schools often lack formal training in ethical issues, such as informed consent, leading to learning opportunities that typically arise through an informal or implicit curriculum [30]. In fact, several medical ethicists highlight a gap between the bioethical theory typically taught in formal university settings and the ethics encountered in clinical practice [6].

Among the reasons some students provide for not obtaining consent is considering themselves part of the medical team, being apprehensive about the evaluation of their performance by house staff [26], or even the belief that patients implicitly consent to the involvement of medical students in their care when they consent to treatment at a teaching hospital [24]. Another explanation for students to not clearly disclose their lack of experience to patients is fearing that fully informed patients may not allow them to participate in all aspects of the patients’ care [19]. This is viewed by medical students as a restriction of their opportunities to improve their clinical skills, which suggests that the teaching hospital’s primary focus may be on medical training, potentially diminishing other considerations such as respect for patient autonomy. Lastly, students may have been under the impression that this practice was acceptable because the staff responsible for their training perpetuated it [26].

Understanding these perceptions is crucial for addressing students’ concerns regarding consent, integrating these discussions more effectively into medical education curricula, and maintaining the integrity of patient autonomy within the realm of medical education [24]. Encouraging and inspiring students to uphold exemplary ethical conduct requires enhancing the quality of education and integrating relevant and pragmatic content relating to ethical matters [23].

When it comes to the role of clinical teachers, they are responsible for ensuring that patients are informed that medical students may be involved in their care, and are given the chance to decline care from students [9]. As in numerous aspects of medical education, teachers should set an example by accurately introducing students to patients [26].

Nonetheless, the reported lack of awareness of the National Code of Ethics for Medical Students among more than half of teachers is an important finding of this study. Additionally, considering the significant influence of the hidden curriculum on teaching professionalism, witnessing unethical behavior, particularly in the presence of patients, can have profoundly adverse effects on students [23].

It Is important to mention that obtaining informed consent for the involvement of students in patient care can sometimes also present difficulties for attending teachers [17]. Actually, this study’s findings regarding teachers’ perspectives raise significant concerns about the quality of medical education and patient care within the educational setting, as a high percentage of tutors perceived the current consultation dynamics as inadequate for competency acquisition. The identified limitations, including lack of time, high numbers of students and possible damage to the doctor–patient therapeutic relationship, are critical barriers to effective learning and patient-centered care.

However, when it comes to the fear of damaging the doctor–patient relationship, the literature demonstrates that the overall satisfaction level of patients with their care remains consistent regardless of whether they consulted solely with their physicians or in the presence of medical students. Furthermore, clinical educators at university teaching hospitals should allocate dedicated time for patients without the presence of medical students. This ensures that issues that may be uncomfortable to address in front of students can be discussed openly [2].

These challenges may not only harm students’ ability to develop essential clinical skills, but also compromise the quality of patient interactions and overall healthcare delivery. Additionality, when explicit consent for student involvement in patient care is not obtained, medical educators miss a fundamental opportunity to encourage students to be ethically sensitive and responsible professionals [17].

In this study, almost half of teachers asked for the patients’ consent when the students were already present. Addressing this finding is crucial, as asking for consent after a patient has undressed or in the presence of students may make it more challenging for the patient to refuse. If the doctor feels that the presence of a student in the consultation room at the time of requesting consent for their presence during the consultation may result in the patient feeling pressured to accept, a reasonable alternative would be to go to the waiting room and ask the patient if they would like to have students present during the consultation that day. This way, the patient will feel freer to request to be alone with their doctor. Therefore, teachers play a role in ensuring that consent is obtained without any hint of pressure [21].

On the other hand, when comparing students and teachers’ perceptions, there appears to be a discrepancy between teachers’ self-reported practices concerning patient consent and students’ perceptions, as the majority of teachers claimed to always introduce students to the patient and to always request consent from patients for student presence, while only a fraction of students reported this to be the case. This disparity suggests a misalignment in educators’ and learners’ communication or understanding, which underlines the importance of maintaining a clear and transparent dialogue regarding ethical practices in patient care, and of a standard method of disclosure.

Moreover, the difference between students and teachers’ self-reported behaviors on the discussion of technical terms in the patients’ presence reveals a potential gap in professionalism and patient-centered communication. While novice students may believe they are engaging in appropriate behavior by discussing technical aspects of care with their teachers, educators may view this practice differently, considering the potential impact on patient understanding and comfort. This finding highlights the importance of ongoing feedback and mentorship to guide students in developing effective communication skills and maintaining patient-centered care.

As in this study’s results, the literature shows a necessity to improve education for both teachers and medical students concerning their obligations in obtaining and ensuring appropriate consent for medical student involvement in patient care [31].

To achieve this, one study recommended medical students be required to complete an online module on consent before starting their clinical rotations, in order to equip students with the necessary knowledge and confidence to appropriately seek consent from patients [31]. Additionally, students should engage in simulated interactions and small-group discussions to develop the psychosocial skills required for effective patient communication during the informed consent process [8]. It is also essential that students be briefed on the teaching hospital’s policy [25], as well as provided with clear guidelines regarding disclosure and informed consent [20].

In regard to teachers, the literature proposes the implementation of faculty development programs aimed at offering instructors formal training in ethics, as this would enhance their readiness to teach ethics effectively [7]. Another suggestion is that teachers adopt a standardized framework to regularize the participation of students within their care team and promote discussions with patients relating to this matter [24].

This study had a few limitations. Primarily, it depended on students’ and teachers’ recollection of past encounters with patients and how they behaved when requesting informed consent, introducing potential memory bias. Moreover, this study is based on a web-based survey, disseminated through email, which might have been influenced by self-selection bias. In addition, the research was conducted exclusively at a single medical school and teaching hospital in Portugal, suggesting the necessity for broader and multicenter studies to validate these findings. While our study was carried out in a single medical school in Portugal, we provided a characterization of our sample, which is crucial for understanding the context and applicability of our findings. Lastly, the sample size is small; while we recognize the limitations posed by a smaller sample, we believe that the findings still offer valuable insights that can stimulate further research and discussions.

## 5. Conclusions

In conclusion, this study examines the delicate balance between meeting the educational needs of medical training and its potential benefits for patients, while upholding ethical principles such as beneficence and autonomy. It also provides medical students and their teachers key data relating to the importance of obtaining patients’ consent to be involved in medical education.

Overall, these findings highlight an urgent need for the restructuring of ethical values in medical education through standardized procedures, in order to ensure optimal learning experiences and teaching environments for both students and teachers. This reorganization requires strategic interventions at institutional levels that could allow for a simultaneous high quality of patient care and clinical training. It is important to consider the role of medical institutions and regulatory organizations in promoting a strong ethical culture in medical education. This may include developing and implementing clear policies and ethical guidelines, as well as creating support systems and resources to address complex ethical issues that arise in the clinical setting.

Additionally, it is crucial to recognize that ethical challenges in medical education are dynamic and multifaceted, requiring a continuous and adaptive approach. This includes constantly updating medical schools’ educational curricula to reflect advances in medical ethics, as well as continually training students on emerging ethical issues in medical practice.

Finally, in addition to promoting respect for ethical principles, it is essential to cultivate in students and teachers a deep understanding of the ethical impact of their actions on the patient’s well-being and the integrity of the medical profession as a whole.

## Figures and Tables

**Table 1:** Students and teachers’ survey answers.

	Students’ Answers		Teachers’ Answers	
	*n*	(%)	*n*	(%)
Teachers always ask the patient for consent to be in the presence of students.	65	(28)	51	(81)
When asking for consent, do teachers do so before students enter the room or in the presence of students?				
Never ask for consent	10	(4)	1	(2)
Ask in the absence of students	29	(13)	28	(49)
Ask in the presence of students	193	(83)	28	(49)
Teachers always introduce students to the patient.	83	(36)	50	(88)
Have you ever felt that the patient was uncomfortable with the presence of students?				
Never	46	(20)	7	(11)
Already happened	179	(77)	44	(77)
It happens often	7	(3)	6	(12)
How do you consider the number of students in relation to the discomfort caused to the patient in the medical appointment?				
None	3	(1)	9	(16)
Little	49	(21)	24	(42)
Moderate	128	(55)	18	(32)
Very	52	(23)	6	(11)
Do you discuss with your teachers/students using medical jargon in the presence of patients?				
Never	33	(14)	16	(28)
Already happened	119	(51)	31	(54)
It happens often	80	(35)	10	(18)

**Table 2:** Students’ survey answers by academic year.

	4th Year		5th Year		6th Year		
	*n*	(%)	*n*	(%)	*n*	(%)	*p* *
Teachers always ask the patient for consent to be in the presence of students.	31	(34)	19	(29)	15	(20)	0.142
When asking for consent, do teachers do so before students enter the room or in the presence of students?							**0.026**
Never ask for consent	5	(6)	0	(0)	5	(7)	
Ask in the absence of students	16	(17)	9	(14)	4	(5)	
Ask in the presence of students	71	(77)	56	(86)	66	(88)	
Teachers always introduce students to the patient.	34	(37)	26	(40)	23	(31)	0.488
Have you ever felt that the patient was uncomfortable with the presence of students?							0.630
Never	19	(21)	14	(22)	13	(17)	
Already happened	72	(78)	48	(74)	59	(79)	
It happens often	1	(1)	3	(5)	3	(4)	
How do you consider the number of students in relation to the discomfort caused to the patient in the medical appointment?							0.145
None	0	(0)	0	(0)	3	(4)	
Little	23	(25)	10	(15)	16	(21)	
Moderate	46	(50)	38	(59)	44	(59)	
Very	23	(25)	17	(26)	12	(16)	
Do you discuss with your teachers using medical jargon in the presence of patients?							**<0.001**
Never	8	(9)	7	(11)	18	(24)	
Already happened	40	(43)	36	(55)	43	(57)	
It happens often	44	(48)	22	(34)	14	(19)	
Do you always present yourself as a student?	77	(84)	58	(89)	65	(87)	0.606
When presenting yourself, do you mention your academic year?	43	(47)	36	(55)	44	(59)	0.278
Do you inform the patient about the procedure that will be used, explaining its objective and clarifying doubts?							0.143
Never	2	(2)	1	(2)	0	(0)	
Already happened	22	(24)	21	(32)	13	(17)	

* *p*-value < 0.05. Bold: significant *p*-values.

## Data Availability

The datasets used and analyzed during the current study are available from the corresponding author on reasonable request.
